# Wiederaufnahmeraten von Rückenschmerzpatienten an einer Universitätsklinik nach primär konservativer stationärer Therapie

**DOI:** 10.1007/s00132-020-04043-y

**Published:** 2020-11-28

**Authors:** Florian Ihde, Robert Lenz, Wolfram Mittelmeier, Katrin Osmanski-Zenk

**Affiliations:** Orthopädische Klinik und Poliklinik, Universitätsmedizin Rostock, Doberaner Str. 142, 18057 Rostock, Deutschland

**Keywords:** Nachsorge, Notaufnahme, Hospitalisation, Nukleotomie, Retrospektive Studie, Aftercare, Emergency room, Hospitalization, Nucleotomy, Retrospective study

## Abstract

**Hintergrund:**

Die Patientenzahlen stationärer Versorgung von Rückenschmerzen steigen zunehmend, da die aktuellen Strukturen der ambulanten Versorgung den Bedarf nicht adäquat decken können. Die Infrastruktur des Maximalversorgers sichert zwar einerseits eventuelle Notfallversorgungen und bildgebende Verfahren ab, ist aber andererseits nicht auf die Ersatzleistung für die ambulante Versorgung ausgerichtet.

**Ziel der Arbeit:**

Analyse der Wiederaufnahmeraten stationär primär konservativ versorgter Rückenschmerzpatienten.

**Material und Methode:**

In der retrospektiven Studie wurde an einer universitären orthopädischen Klinik die Wiederaufnahmerate von Rückenschmerzpatienten innerhalb von 6 Monaten untersucht, die notfallmäßig stationär eingewiesen und dort primär konservativ behandelt wurden. Der Untersuchungszeitraum betrug 2 Jahre mit einer Nachbeobachtung von 6 Monaten. Es erfolgte die Auswertung von 413 Patienten.

**Ergebnisse:**

Nach primär konservativer stationärer Therapie wurden 17,9 % der Patienten erneut stationär aufgenommen. Bis zur ersten Wiederaufnahme dauerte es 25 (±33,25) Tage und bis zur zweiten weitere 25,9 (±31,99) Tage. Rentner werden signifikant häufiger stationär aufgenommen, und in der Regel konservativ behandelt. 66,8 % der Vorstellungen erfolgten notfallmäßig ohne Einweisungsschein.

**Diskussion:**

Die Wiederaufnahmen nach primärer konservativer stationärer Therapie sind relativ hoch. Bei straffem Management mit zügigem diagnostischem Vorgehen und zielgerichteten Nachbehandlungsstrategien kann die Rückführung des Patienten in die ambulante Versorgung in den meisten Fällen realisiert werden. Wiederaufnahmen erfolgen zur operativen Versorgung, oder aber ungeplant bei gescheiterter konservativer, ambulanter Therapie.

## Abstract (en)

**Background:**

The number of patients receiving inpatient treatment for back pain is increasing, as the current structures of outpatient care cannot meet the demand adequately. Although the infrastructure of the maximum care provider ensures possible emergency care and imaging procedures on the one hand, it is not geared to providing replacement services for outpatient care on the other.

**Objectives:**

Analysis of the readmission rates of primarily conservatively treated inpatients with back pain.

**Materials and methods:**

In this retrospective study, the recovery rate of patients with back pain who were admitted as emergency inpatients and treated primarily conservatively as inpatients was investigated within 6 months at a university orthopaedic clinic. The study period was 2 years with a follow-up of 6 months. 413 patients were evaluated.

**Results:**

After primarily conservative therapy, 17.9% of the patients were readmitted to hospital. It took 25 (±33.25) days until the first readmission and 25.9 (±31.99) days until the second readmission. Pensioners were admitted to hospital significantly more often but were treated mainly conservatively during their stays; 66.8% of the presentations were emergencies without referral.

**Conclusions:**

Readmission after primarily conservative inpatient treatment is relatively high. In most cases, the return of the patient to outpatient care can be achieved by tight management with a rapid diagnostic procedure and targeted aftercare strategies. The patient may return to outpatient care for surgical treatment or, unplanned, due to failed conservative, outpatient treatment.

## Hintergrund und Fragestellung

Rückenschmerzen gehören zu den Volksleiden in Deutschland, 85,5 % der Bevölkerung sind davon in ihrem Leben betroffen [10]. Dies führt zu hohen gesellschaftlichen Kosten. Je nach Schweregrad der Erkrankung reichen die Folgen von kurzer Arbeitsunfähigkeit bis hin zur Frühverrentung und der damit verbundenen Einschränkung der Lebensqualität [4].

Der Anspruch der modernen Bevölkerung bezüglich Freizeitgestaltung und Selbstständigkeit bis ins hohe Alter nimmt zu. Aufgrund der gesellschaftlichen Veränderungen, mit der Tendenz zu mehr Alleinlebenden [8], können die Patienten nicht in jeder Akutsituation auf familiäre Hilfe zurückgreifen. Derartige Umstände treten nicht selten außerhalb der Sprechzeiten ein, sodass sich die Patienten notfallmäßig in der Notaufnahme bzw. Poliklinik eines Krankenhauses vorstellen. Die Erwartung an die Ärzte im Sinne einer unverzüglichen Bereitstellung diagnostischer und therapeutischer Dienstleistungen ist hierbei sehr groß. Es bietet sich aber auch die Chance an, als Notfallpatient schneller diagnostiziert und behandelt zu werden, um Wartezeiten und Gänge über Hausarzt und Fachärzte zu vermeiden.

Krankenhausnotaufnahmen sind überlastet durch Patienten, die keine echten, dringenden Notfälle darstellen [9]. Im orthopädischen Fachbereich überschreiten Rückenschmerzpatienten im Hinblick auf Diagnostik und stationäre Verweildauer diesbezüglich schnell die Grenze der Wirtschaftlichkeit. Die Realität zeigt, dass ein wesentlicher Anteil der als dringend stationär behandlungsbedürftig eingeschätzten Patienten häufig entweder nicht ausreichend schmerztherapeutisch eingestellt ist, nicht entsprechend diagnostiziert wurde oder lediglich unzureichend häuslich bzw. anderweitig versorgt wird.

Der Kostendruck in Krankenhäusern ist groß. Die konservative stationäre Therapie und ambulante Notfallversorgung ist im durchgängigen, leistungsorientierten und pauschalierenden Vergütungssystem für Krankenhäuser (German Diagnosis Related Groups [G-DRG]) bzw. nach dem einheitlichen Bewertungsmaßstab nicht adäquat vergütet und oft nicht profitabel [3].

Ziel dieser Arbeit ist es, die Patientenpopulation von Rückenschmerzpatienten exemplarisch an einer orthopädischen Universitätsklinik hinsichtlich wesentlicher Patientendaten sowie Therapie- und Diagnostikaufwand im Rahmen einer initialen konservativen stationären Therapie zu prüfen. Des Weiteren wurden die Quoten der Wiedervorstellungen nach initialer konservativer stationärer Therapie untersucht.

## Material und Methoden

In der retrospektiven Studie wurde überprüft, wie oft primär konservativ stationär behandelte Rückenschmerzpatienten an der betreffenden Universitätsklinik innerhalb von 6 Monaten erneut stationär aufgenommen wurden.

Der definierte Untersuchungszeitraum der Studie betrug 2 Jahre (01.01.2013 bis 31.12.2014) mit einer Nachbeobachtungszeit für die Wiedervorstellung von 6 Monaten (bis zum 30.06.2015).

In die Studie wurden die stationär aufgenommenen Rückenschmerzpatienten eingeschlossen, die während des ersten stationären Aufenthaltes nicht operativ behandelt wurden, somit also einer primär konservativen Behandlung folgten. Primär ausgeschlossen wurden alle Patienten, die während des Untersuchungszeitraums innerhalb von 6 Monaten vor dem ersten konservativen stationären Aufenthalt einen stationären Aufenthalt mit operativer Versorgung aufgrund ihrer Rückenschmerzen hatten.

**Figure 1:**
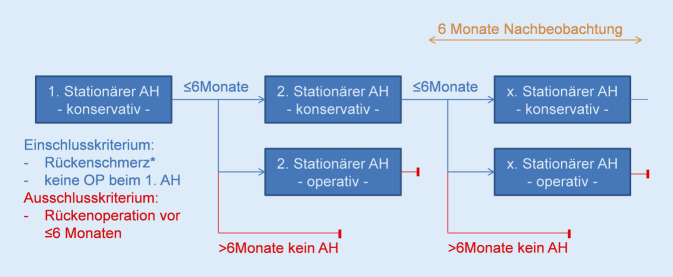

Im Untersuchungszeitraum wurden nur Folgeaufenthalte von den Patienten erfasst, die innerhalb von 6 Monaten (Abb. 1) wieder notfallmäßig aufgenommen wurden. Sobald der Patient einen operativen oder innerhalb von 6 Monaten keinen Folgeaufenthalt hatte, wurden die weiteren Aufenthalte nicht mehr untersucht.

Die statistische Auswertung erfolgte mit dem Statistikprogramm SPSS 22.0 (IBM, Armonk, NY, USA) und die Erstellung der Diagramme mit Microsoft Excel. Für die Überprüfung von Unterschieden zwischen allen Gruppen wurde der Chi-Quadrat-Test bzw. Kruskal-Wallis-H-Test genutzt. Bei nachgewiesener Signifikanz wurden die einzelnen Gruppen mit dem Chi-Quadrat-Test bzw. Mann-Whitney-U-Test miteinander verglichen. In der Studie lag ein signifikanter Unterschied vor, wenn *p* ≤ 0,05 war.

**Figure 2:**
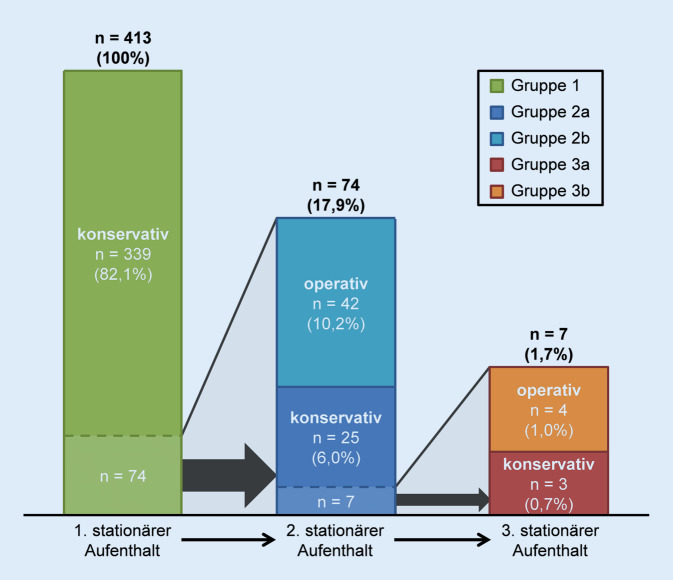

1298 aufeinanderfolgende Patienten mit der Einweisungsdiagnose Rückenschmerzen wurden auf ihre Eignung zum Einschluss in die Studie geprüft. Ausgeschlossen wurden hier Patienten (*n* = 885), bei denen Rückenschmerzen als Nebendiagnose (*n* = 568) erfasst waren oder eine operative Versorgung (*n* = 317) bereits beim ersten Aufenthalt erfolgte. 413 Patienten erfüllten die Einschlusskriterien, wurden in Gruppen aufgeteilt und entsprechend der Zielkriterien untersucht (Abb. 2):

- Gruppe 1 (*n* = 339): ein konservativer stationärer Aufenthalt
- Gruppe 2a (*n* = 25): zwei konservative stationäre Aufenthalte
- Gruppe 2b (*n* = 42): ein konservativer stationärer Aufenthalt und ein operativer
- Gruppe 3a (*n* = 3): drei konservative stationäre Aufenthalte
- Gruppe 3b (*n* = 4): zwei konservative stationäre Aufenthalte und ein operativer

## Ergebnisse

Im Erfassungszeitraum war von 1298 stationär aufgenommen Patienten mit der Diagnose Rückenschmerz bei 730 Patienten der Rückenschmerz als Hauptdiagnose verzeichnet. 317 Patienten wurden – Notfallaufnahmen eingeschlossen – operativ versorgt.

Ältere Patienten und Frauen werden häufiger stationär aufgenommen. Ein Großteil der Patienten leidet an Übergewicht (Tab. 1). Die Patientencharakteristika haben keinen signifikanten Einfluss auf die Wiederaufnahmerate.

**Table 1:** 

Parameter	Gruppe 1	Gruppe 2a	Gruppe 2b	Gruppe 3a	Gruppe 3b	Total
*n*	339	25	42	3	4	413
Alter (Jahre)	63,4 (±16,8)	68,0 (±18,4)	64,3 (±14,45)	71,3 (±8,0)	55,8 (±20,7)	63,8 (±16,7)
Geschlecht, Männer	151 (44,5 %)	13 (52,0 %)	17 (40,5 %)	1(33,3 %)	1 (25,0 %)	183 (44,3 %)
BMI	27,5 (±5,3)	29,7 (±9,4)	28,2 (±4,9)	27,4 (±4,0)	24,3 (±3,2)	27,7 (±5,6)

*BMI* Body-Mass-Index

413 Patienten wurden im gleichen Zeitraum primär konservativ über durchschnittlich 3,1 ± 1,8 Tage stationär behandelt.

17,9 % der Patienten wurden mindestens einmal stationär wiederaufgenommen. 88,8 % der konservativ therapierten Patienten wechselten den Therapiearm nicht und wurden durchgehend konservativ behandelt. Lediglich 10,2 % der primär konservativ behandelten Patienten wechselten bei der ersten und noch 1,0 % der Patienten im Rahmen der zweiten Wiederaufnahme in den operativen Therapiearm (Abb. 2). Im Durchschnitt dauerte es 25 (±33,2) Tage bis zur ersten Wiederaufnahme und bis zur zweiten weitere 25,9 (±31,9) Tage, vom Tag der vorherigen Entlassung an.

**Figure 3:**
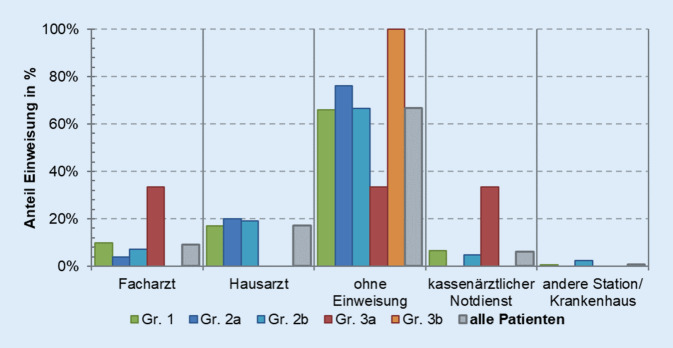

**Figure 4:**
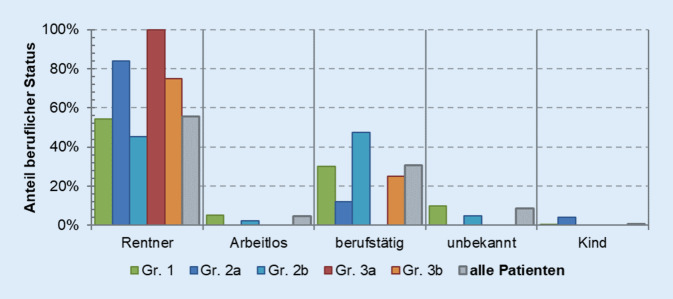

Die Initiierung des ersten stationären Aufenthaltes erfolgte in 66,8 % der Vorstellungen ohne vorausgegangene ärztliche Einweisung in die Klinik (Abb. 3). Andere Einweisungsarten waren in absteigender Reihenfolge über den Facharzt, Hausarzt, Kassenärztlichen Notdienst oder über ein anderes Krankenhaus. Die Mehrheit der aufgenommenen Patienten (55,7 %) wird dabei von älteren Menschen im Rentenalter gestellt, gefolgt von den Berufstätigen (Abb. 4). Diese Gruppe zeichnete sich auch durch eine doppelt so hohe Rate an rein konservativer Behandlung aus (Gruppe 2a).

Als Nebenkriterium wurden die eingeschlossenen Patienten nach radikulären und pseudoradikulären Schmerzen aufgeteilt. Beim ersten Aufenthalt haben 72,9 % der Patienten einen pseudoradikuläres Schmerzbild. Besonders die Gruppen mit rein konservativer Behandlung (1 und 2a) zeichneten sich durch einen hohen Anteil am pseudoradikulärem Schmerz aus. Die Gruppe 2b hat einen signifikant höheren Anteil an Patienten mit radikulärer Schmerzsymptomatik als Gruppe 1 (*p* < 0,001) und 2a (*p* = 0,005). Vom ersten. zum zweiten Aufenthalt verschiebt sich der Anteil mehr zum radikulären Schmerz. Besonders der Anteil bei der operativen Gruppe 2b steigt von 54,8 % auf 76,2 %.

**Figure 5:**
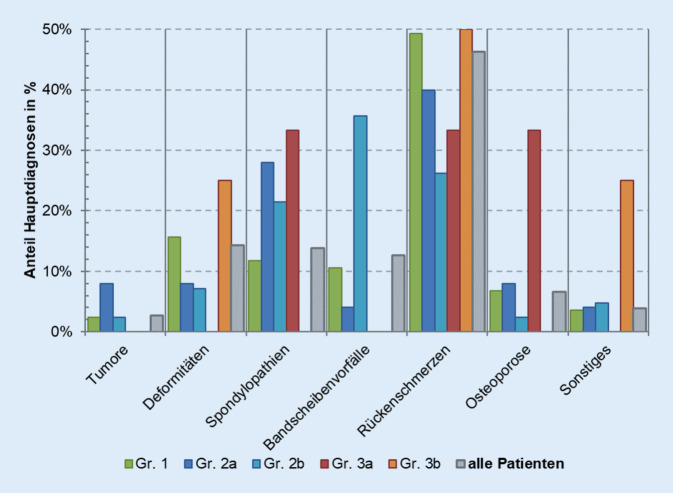

**Figure 6:**
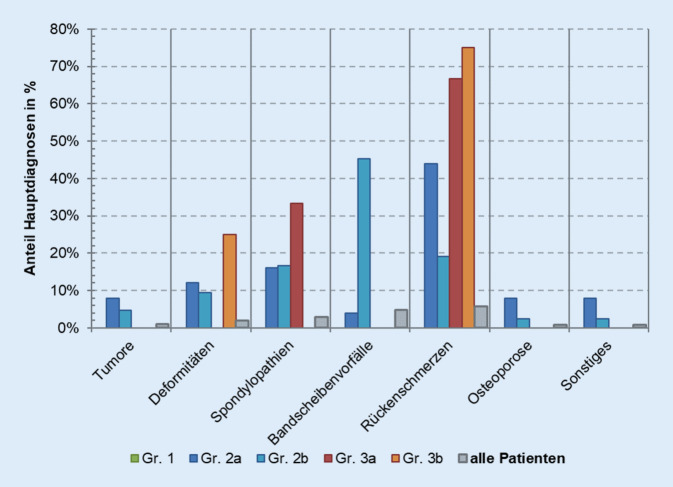

Eine weitere Analyse erfolgte hinsichtlich der Differenzierung der unterschiedlichen Hauptdiagnosen. Die Einteilung erfolgte nach unspezifischen Rückenschmerzen und spezifischen Diagnosen wie Bandscheibenvorfälle, Spondylopathien, Deformitäten, Tumoren und sonstigen Ursachen. Die Gruppe der Patienten mit Tumoren setzt sich hauptsächlich aus Betroffenen mit sekundären Neubildungen an der Wirbelsäule zusammen. Diese Gruppe ist relativ klein (2,7 %), da wenige akut konservativ behandelt werden. Aber im Rahmen eines bekannten Therapiekonzeptes oder zur symptomatischen Schmerztherapie ist die konservative Therapie möglich. Die Hauptdiagnosen weisen sehr signifikante Unterschiede zwischen den einzelnen Gruppen auf (*p* = 0,002). So stellen Patienten mit unspezifischen Rückenschmerzen den größten Anteil derer mit rein konservativer stationärer Behandlung während des ersten Aufenthaltes. Besonders unterscheidet sich Gruppe 2b von der Gruppe 1 (*p* < 0,001). Zwischen den Gruppen 1 und 2a (*p* = 0,133) und den Gruppen 2a und 2b (*p* = 0,121) besteht keine Signifikanz (Abb. 5). Beim zweiten Aufenthalt präzisiert sich die Hauptdiagnose der operativ versorgten Gruppe 2b vom unspezifischen Rückenschmerz (M54 nach ICD-10) zum Bandscheibenvorfall (M51 nach ICD-10) (Abb. 6).

**Figure 7:**
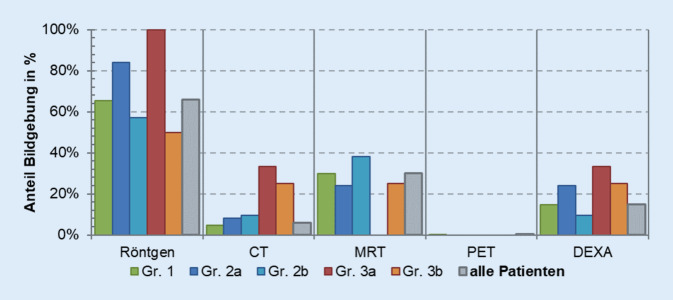

**Figure 8:**
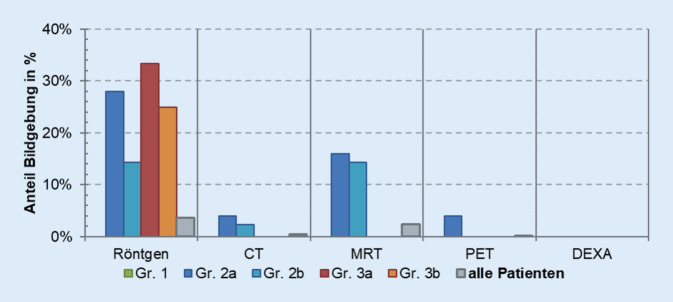

Eine weitere Differenzierung der Gruppen erfolgte nach dem diagnostischen Aufwand. 65,9 % der Patienten erhalten eine Röntgenuntersuchung eines spezifischen Abschnittes der Wirbelsäule beim ersten Aufenthalt. 30 % der Patienten erhielten im Rahmen des ersten stationären Aufenthaltes eine kernspintomographische Untersuchung der Wirbelsäule. Dabei bestand keine Signifikanz zwischen den einzelnen Gruppen. Weitere schnittbildgebende Verfahren wie die CT oder PET-CT spielten keine wesentliche Rolle (Abb. 7). Eine Messung der Knochendichte wurde in 15 % der Fälle durchgeführt. Bei den weiteren Aufenthalten sinkt der diagnostische Aufwand, da die Patienten in der Klinik bekannt sind (Abb. 8).

## Diskussion

Die vorliegende Studie beschreibt die Versorgung von Rückenschmerzpatienten an einer orthopädischen Universitätsklinik vor allem im Hinblick auf die Entwicklung nach initialer konservativer Therapie im stationären Bereich. Mit einer ermittelten Wiederaufnahmerate von 17,9 % der Patienten konnten vergleichbare Werte anderer Studien bestätigt werden. Die Vergleichbarkeit gestaltet sich allerdings bei allgemein knapper aktueller Datenlage schwierig. In der Studie von Nüssler et al. von 2006 mit 916.137 untersuchten Patienten einer Krankenkasse zeigte sich eine Wiederaufnahmerate von 12,7 % innerhalb eines Monats [7]. In einer anderen Studie von Swart et al. von 2005 mit knapp 860.000 untersuchten Patienten betrug die Wiederaufnahmerate innerhalb von 30 Tagen 18,5 % und insgesamt 43,4 % [11]. In beiden Studien wurden alle Krankheitsbilder miteingeschlossen, sowie die Nachverfolgung in verschiedene Krankenhäuser, was auch die höhere Wiederaufnahmerate insgesamt erklärt.

Die Patienten weisen die gleichen Charakteristika wie vergleichbare Rückenschmerzpatienten auf. Auch eine Studie von Neuhauser et al. von 2005 zeigt, dass die Prävalenz bis zum Beginn des 60. Lebensjahr am höchsten ist und Frauen einen größeren Anteil ausmachen [6]. Die Unterschiede sind nicht allein durch verschiedene berufliche Belastungen zu erklären.

Im Mittel beträgt der BMI der Patienten 27,7 (±5,6). Damit zeigt diese Studie, wie auch eine Untersuchung von Leboeuf-Yde et al., dass Übergewicht (BMI >25) ein Risikofaktor für Rückenschmerzen ist [5].

Für die beschriebene hohe Quote der Wiederaufnahmen gibt es unterschiedliche mögliche Gründe: Einer der wesentlichen Gründe ist mit hoher Wahrscheinlichkeit eine unzureichende ambulante fach- und/oder hausärztliche Anbindung nach Entlassung aus dem Krankenhaus. Nach initialer Aufnahme im Krankenhaus kann durch kurzfristige komplexe Behandlung mit intravenöser Schmerzmedikation und Physiotherapie sowie Entlastung vom psychosozialen Umfeld eine rasche Beschwerdebesserung erzielt werden, allerdings wird dies in vielen Fällen jedoch nicht in ausreichendem Maße fortgesetzt, sodass es zu entsprechenden Rezidiven kommt. Hier spielt neben der rein medizinischen Behandlung gemäß den AWMF-Leitlinien zur Behandlung des unspezifischen Rückenschmerzes möglicherweise auch die Patientenedukation eine Rolle [1]. So äußert sich häufig eine wesentliche Motivation der Patienten, sich stationär behandeln zu lassen, in der Annahme einer angeblich schnelleren und umfassenderen Diagnostik, vor allem seitens der Bildgebung mittels Röntgen und MRT. Eine Studie der Arbeitsgruppe Schmiedhofer et al. von 2017 zeigt, dass eine Hauptmotivation von Patienten mit akuten Rückenschmerzen ohne notfallmedizinischen Behandlungsbedarf, die schnelle Verfügbarkeit multidisziplinärer Diagnostik ist [9]. Die Notwendigkeit einer weiteren Aufnahme reduziert sich nach initial erfolgter, vor allem apparativer, Diagnostik, zumal auch niedergelassene Kollegen zugunsten ihres eigenen Budgets Patienten gezielt zur Diagnostik in die stationäre Behandlung einweisen. Auch die Patienten die leitliniengerecht keine unnötige Diagnostik erhalten haben, zeichneten sich dann durch eine geringere Wiederaufnahmerate aus, wenn sie eine verständliche Aufklärung über ihr Krankheitsbild erhalten haben und ihnen der Irrglaube genommen wurde, alles sofort zu bekommen.

Ein möglicher Selektionsfehler kann im monozentrischen Untersuchungsdesign liegen. Eine Reduktion der Wiederaufnahmerate ist prinzipiell durch eine Folgebehandlung in einem anderen Krankenhaus möglich. Allerdings wird erfahrungsgemäß nach Initialversorgung beim Maximalversorger dieser in der Region auch wieder aufgesucht.

Eine weitere Einschränkung ist, dass es unklar bleibt, ob Patienten vor dem Untersuchungszeitraum innerhalb von 6 Monaten schon woanders operativ stationär versorgt wurden und somit ausgeschlossen werden mussten. Die körperliche Untersuchung und Anamnese zeigen, dass dieser Einfluss zu vernachlässigen ist. Aber ungenaue Patientenangaben lassen ein zuverlässiges Anwenden der Ausschlusskriterien nicht zu, sodass wir dies nicht als Ausschlusskriterium nutzen konnten.

Ein signifikantes Ergebnis dieser Studie ist, dass sich Rentner wiederholt stationär vorstellen, aber in den meisten Fällen rein konservativ behandelt werden. Diesbezüglich müsste geklärt werden, ob und warum für dieses Patientenkollektiv die ambulante Versorgung ungenügend ist. In weiteren Untersuchungen ist zu analysieren, inwieweit dies durch individuelle Probleme, wie zum Beispiel Schmerzen, begründet ist oder ob regionale strukturelle Gegebenheiten die primäre Vorstellung in der Notaufnahme bevorzugen lassen. Nicht untersucht wurde, ob grundsätzlich eine Operationsindikation bestand, diese jedoch vom Patienten abgelehnt wird, womöglich durch mediale Verunsicherungen, oder aufgrund von Begleiterkrankungen nicht durchführbar ist.

Ein weiterer interessanter Aspekt dieser Studie ist die Tatsache, dass sich 66,8 % der Patienten ohne Einweisung durch einen niedergelassenen Arzt beim ersten Aufenthalt in der Notaufnahme vorstellen. Hier zeigt sich deutlich eine mangelnde Kontaktaufnahme zur Niederlassung, welche vom Patienten häufig mit einer unzureichenden Terminverfügbarkeit begründet wird. Dahingegen zeigt die Arbeit von Tille et al. jedoch, dass es für 80 % der Patienten innerhalb von 3 Tagen einen Termin beim Hausarzt geben kann und eine Akutversorgung, auch wie in dieser Studie beschrieben, zeitnah möglich ist. Die Wartezeiten bei einem Facharzt können indessen durchaus länger sein [12]. Die Studie von Schmiedhofer et al. belegt, dass die Unabhängigkeit von Öffnungszeiten, das Wegfallen der Terminorganisation und die höchste Behandlungsqualität eine der größten Motivationen für die Vorstellung in der Notaufnahme sind [9]. In Zukunft muss geklärt werden, warum die Patienten in Akutsituationen nicht den Hausarzt aufsuchen, sondern sich sofort in der Notaufnahme einer Klinik vorstellen und wie dieses Verhalten der Patienten verändert werden kann, um Kosten und Personalressourcen zu schonen. Die vorliegenden Daten wurden noch vor Inkrafttreten des Gesetzes zur Stärkung der Versorgung in der gesetzlichen Krankenversicherung (GKV-Versorgungsstärkungsgesetz) erhoben [2]. Die Auswirkung der darin geforderten Einrichtung von Terminservicestellen auf die Versorgungsqualität ist derzeit noch nicht bekannt.

Ziel dieser Gesetzgebung ist eine suffiziente Versorgung durch Fachärzte. So sollte im Fachbereich Orthopädie und Unfallchirurgie ein großer Teil der Patienten ambulant versorgt werden können. Einweisungen ins Krankenhaus sollten dementsprechend Notfällen vorbehalten sein. Auch der Allgemeinmediziner kann zur ambulanten Therapie beitragen. Die Zusammenarbeit der verschiedenen ambulanten Fachrichtungen sollte in Zukunft verbessert werden. In Akutsituationen kann der Hausarzt in der Regel eine adäquate Therapie beginnen und die Zeit bis zum Facharzttermin überbrücken, falls dieser überhaupt nötig ist. Außerdem kann der Hausarzt die Behandlung von chronischen Rückenschmerzen nach einem entsprechenden Therapieplan vom Facharzt fortführen.

Dennoch bleibt die Notaufnahme ein essenzieller Bestandteil des Gesundheitssystems und die Herausforderungen unter den vielen unberechtigten Vorstellungen, den ernsthaften Kranken zu erkennen, werden trotz gesetzlicher Regularien fortbestehen.

## Schlussfolgerung

Die Wiederaufnahmen sind nach primärer konservativer stationärer Therapie relativ hoch. Die stationäre Behandlung von Rückenschmerzen und die Rückführung in eine ambulante Therapie sind aufgrund von strukturierten Therapiekonzepten gut umsetzbar. Dennoch sind viele dieser Behandlungsmodalitäten auch ambulant umsetzbar, sodass mit geeigneten gesundheitspolitischen Konzepten die Behandlung von Rückenschmerzen im niedergelassenen Bereich möglich sein sollte, damit überflüssige stationäre Aufnahmen vermieden werden können.

In Zukunft muss untersucht werden, warum die Patienten nicht ausreichend ambulant im kassenärztlichen System versorgt sind und sich in Akutsituationen in die Notaufnahme eines Krankenhauses begeben. Diese sollte lediglich akuten Notfallereignissen zur Verfügung stehen und nicht als Serviceleistung für Patienten, die sich nicht um Termine kümmern und ihre Zeitgestaltung nicht an die Öffnungszeiten der Praxen anpassen möchten [9].

So sollten die ambulanten Versorgungsmöglichkeiten für die Patienten attraktiver gemacht werden, damit die Motivationen für eine Vorstellung in der Notaufnahme sinken. Den Patienten muss aber auch ein transparenter Diagnostik- und Therapiealgorithmus zur Verfügung gestellt werden, der wie im Krankenhaus schnelle, kompetente Entscheidungen und kurze Wege ermöglicht.

## Fazit für die Praxis

- Die Wiederaufnahmerate von Patienten mit Rückenschmerzen ist relativ hoch.
- Bei rein konservativem Therapieansatz ist eine verbesserte ambulante Anbindung anzustreben.
- Vor allem für ältere Patienten sollte der Zugang zu niedergelassenen Ärzten verbessert werden, um unnötige Krankenhausaufnahmen zu vermeiden.
- Diagnostische und therapeutische Ressourcen sollten daher im niedergelassenen Bereich ausgebaut werden, um den Druck von den Notaufnahmen zu nehmen.

## Abkürzungen

AWMF: Arbeitsgemeinschaft der Wissenschaftlichen Medizinischen Fachgesellschaften
BMI: Body-Mass-Index
G‑DRG: German Diagnosis Related Groups
GKV: Gesetzliche Krankenversicherung
ICD: International Statistical Classification of Diseases and Related Health Problems
