# Erratum zu: Wiederaufnahmeraten von Rückenschmerzpatienten an einer Universitätsklinik nach primär konservativer stationärer Therapie

**DOI:** 10.1007/s00132-021-04170-0

**Published:** 2021-09-30

**Authors:** Florian Ihde, Robert Lenz, Wolfram Mittelmeier, Katrin Osmanski-Zenk

**Affiliations:** Orthopädische Klinik und Poliklinik, Universitätsmedizin Rostock, Doberaner Str. 142, 18057 Rostock, Deutschland

**Erratum zu:**

**Orthopäde 2020**

10.1007/s00132-020-04043-y

Der Artikel *Wiederaufnahmeraten von Rückenschmerzpatienten an einer Universitätsklinik nach primär konservativer stationärer Therapie *von Florian Ihde, Robert Lenz, Wolfram Mittelmeier, Katrin Osmanski-Zenk wurde ursprünglich am 28. November 2020 ohne „Open Access“ online auf der Internetplattform des Verlags publiziert. Die Autoren haben sich jedoch nachträglich für eine „Open Access“-Veröffentlichung entschieden. Das Urheberrecht des Artikels wurde deshalb 06. Mai 2021 in ©The Author(s) 2020 geändert.

Der Artikel wird nun unter der Creative Commons Namensnennung 4.0 International Lizenz veröffentlicht, welche die Nutzung, Vervielfältigung, Bearbeitung, Verbreitung und Wiedergabe in jeglichem Medium und Format erlaubt, sofern Sie den/die ursprünglichen Autor(en) und die Quelle ordnungsgemäß nennen, einen Link zur Creative Commons Lizenz beifügen und angeben, ob Änderungen vorgenommen wurden.

